# To Approach or Avoid: An Introductory Overview of the Study of Anxiety Using Rodent Assays

**DOI:** 10.3389/fnbeh.2020.00145

**Published:** 2020-08-26

**Authors:** Mimi La-Vu, Brooke C. Tobias, Peter J. Schuette, Avishek Adhikari

**Affiliations:** Department of Psychology, University of California, Los Angeles, Los Angeles, CA, United States

**Keywords:** elevated plus maze (EPM), open field test, anxiety, SSRI (selective serotonin reuptake inhibitor), benzodiazepine

## Abstract

Anxiety is a widely studied phenomenon in behavioral neuroscience, but the recent literature lacks an overview of the major conceptual framework underlying anxiety research to introduce young researchers to the field. In this mini-review article, which is aimed toward new undergraduate and graduate students, we discuss how researchers exploit the approach-avoidance conflict, an internal conflict rodents face between exploration of novel environments and avoidance of danger, to inform rodent assays that allow for the measurement of anxiety-related behavior in the laboratory. We review five widely-used rodent anxiety assays, consider the pharmacological validity of these assays, and discuss neural circuits that have recently been shown to modulate anxiety using the assays described. Finally, we offer related lines of inquiry and comment on potential future directions.

## Introduction

While foraging for food and resources, animals encounter environments with varying degrees of threat. Depending on the level of uncertainty and temporal and spatial proximity to danger, such environments can elicit various behavioral states. Low-threat situations, in which perceived danger is diffuse and uncertain, elicit anxiety and passive avoidance measures while higher-threat situations, in which perceived danger is imminent and well-defined, elicit fear or panic and active avoidance behaviors (Perusini and Fanselow, 2015; Robinson et al., 2019). In this mini-review article, we will focus on the study of anxiety, which in the animal research literature is often defined as a temporary behavioral state induced by low-threat, uncertain situations and accompanied by increased vigilance and risk assessment (Adhikari, 2014; Calhoon and Tye, 2015; Robinson et al., 2019). These behaviors can be adaptive, but patients with anxiety disorders display chronically high levels of anxiety and avoidance measures, generally to a debilitating and maladaptive extent. As one in every 13 U.S. adults develop a generalized anxiety disorder in their lifetime (Ruscio et al., 2017), there exist major clinical and economic implications for identifying neural circuitry and activation patterns that contribute to anxiety. We can study rodent reactions to anxiety-provoking stimuli to better understand the neurobiology underlying anxiety. The recent anxiety literature lacks a broad overview of the current state of the field for young researchers; thus, we aim to deliver a general review geared toward novice undergraduate and graduate researchers entering the study of anxiety. We will discuss how the approach-avoidance conflict informs the use of rodent anxiety assays, the pharmacological validation of these assays, brain regions implicated in anxiety, and related lines of inquiry.

To gauge innate levels of anxiety, researchers have developed behavioral assays that exploit the approach-avoidance conflict facing rodents—balancing the desire to explore novel environments and forage for resources (approach) while evading predators and other potentially harmful threats (avoidance). In general, mice are averse to brightly lit, open spaces and prefer dim, enclosed spaces, presumably to prevent visual detection by predators. Accordingly, anxiety assays are designed to measure the extent to which rodents engage in anticipatory evasion of predatory threat by avoiding bright lights and open spaces. As all assays discussed below exploit the approach-avoidance conflict, various related factors can influence individual performance including, but not limited to, the drive to explore and forage, baseline anxiety, hunger, time of day, social or single housing, and habituation to experimenters. Thus, experiments must be carefully curated to contain appropriate control measures that account for these potential factors (to the best of one’s ability) to enable a clear interpretation of results. Importantly, these assays are more effective without prior exposure.

The primary goal of anxiety research in rodents is to uncover the anatomical and molecular substrates of anxiety to inform the development of novel treatments. Rodents cannot comment on their emotional state, which is arguably the most common method of assessing anxiety in humans. Thus, it is important to maintain that, by employing rodent models, researchers are not necessarily studying the “feeling of anxiety” that often comes to mind when one considers anxiety colloquially. Accordingly, when describing an experimental outcome, it is better practice to explain what is objectively occurring in the context of the behavioral paradigm than to layer on researchers’ interpretations. Stating a mouse is “spending less time in the center of the Open Field” is thus preferable to stating a mouse is “feeling anxious” during a particular trial. Importantly, because each assay probes distinct behaviors and bears unique strengths and limitations, it is most informative to use multiple assays in parallel to provide stronger collective evidence for an anxiety-related phenotype.

### Open Field Test

**Figure 1:**
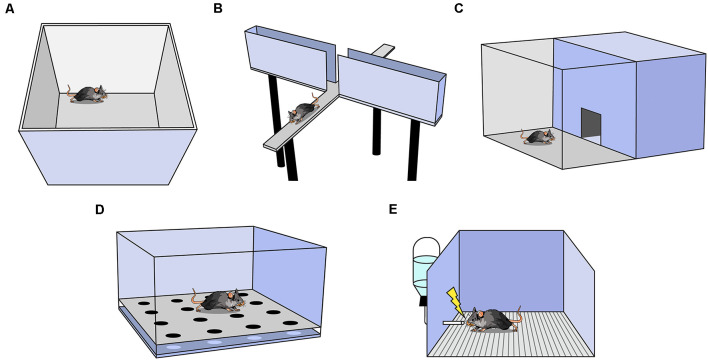
Behavioral assays used to measure anxiety in rodents. **(A)** Open Field Test (OFT). **(B)** Elevated Plus Maze. **(C)** Light-Dark Box. **(D)** Hole Board. **(E)** Vogel Conflict Test.

One of the most commonly used anxiety assays is the Open Field Test (OFT), in which rodents are placed in an empty square or circular arena without a ceiling for 10–15 min (Figure 1A; Seibenhener and Wooten, 2015). The fraction of time spent in the perimeter (thigmotaxis) vs. the center of the context is measured and, as rodents avoid the open center, increased thigmotaxis is interpreted as higher anxiety. The OFT can also be used to measure other behaviors such as locomotion (total distance traveled), velocity, defecation, and latency to enter the center (Seibenhener and Wooten, 2015). Locomotion and velocity are often observed in the OFT before other anxiety assays to assess treatment-induced motor changes that may confound interpretation in other assays. It is important to analyze the ratio of locomotion in the center to total locomotion in the OFT; if an experimental treatment decreases center time, yet also decreases total locomotion, the effect may not be easily interpretable.

### Elevated Plus Maze (EPM) and Related Assays

Another widely utilized assay is the elevated plus maze (EPM; Bertoglio and Carobrez, 2002; Garcia et al., 2011). The EPM is a raised platform in the shape of a plus sign (+), consisting of two enclosed arms with walls intersected by two open arms without walls (Figure 1B). The percent of time spent in the open and enclosed arms as a function of total arm time and the percent of entries into the open and enclosed arms as a function of all arm entries are recorded. Increased percent time and entries into the enclosed arms are interpreted as greater avoidance of elevated, open spaces (Walf and Frye, 2007). For a treatment to be deemed anxiogenic or anxiolytic, one would expect shifts in percent open arm time and percent open arm entries in the same direction. Rodents naturally prefer the enclosed arms, and in a typical 10-min trial, mice spend the majority of the time in the enclosed arms (Komada et al., 2008). The utility of this task falls steeply across multiple exposures as rodents avoid the open arms more in each subsequent exposure, presumably because the animal learns there are no rewards in the potentially dangerous open arms (Walf and Frye, 2007). A variation of the EPM, the elevated zero maze, is ring-shaped, and half of the area of the maze is enclosed by walls (Tucker and Mccabe, 2017). This construction avoids the ambiguous center zone of the standard EPM, as it is unclear if time spent in the center indicates higher anxiety. Another variation is the elevated T-maze which consists of three arms: one enclosed and two open. In this assay, the animal is placed in an open arm and the latency to escape to the enclosed arm is recorded as a measure of panic-related escape (Viana et al., 1994). The animal can also be placed in the enclosed arm and latency to enter an open arm is measured as an assessment of anxiety.

### Light-Dark Box

The Light-Dark Box is another common assay in which mice are placed in an arena with two chambers, one brightly lit and another dark, with free access between the chambers (Figure 1C; Birkett et al., 2011). The number of entries and the amount of time spent in the lit chamber are recorded, and increased avoidance of the lit chamber is interpreted as increased anxiety (Kulesskaya and Voikar, 2014). Other metrics include locomotion and velocity in each chamber, number and length of freezing bouts in each chamber, and latency to enter a chamber (Takao and Miyakawa, 2006). Usually, both light and dark chambers are the same size.

### Hole Board Assay

In the Hole Board Assay, a rodent is placed in an arena similar to the OFT except the floor contains evenly-spaced holes the rodent can poke its head into, referred to as head-dipping (Figure 1D; Brown and Nemes, 2008). Animals who head-dip less into the holes are considered more anxious (Brown and Nemes, 2008). This test is commonly used as a measure of exploratory and repetitive behaviors (Moy et al., 2008).

### Vogel Conflict Test

This assay uses the punishment of a conditioned response to test anxiety (Figure 1E; Millan and Brocco, 2003). It is a learned approach-avoidance task; therefore, it is unlike the previously mentioned assays which rely on innate approach-avoidance behaviors. Rodents are water-deprived, then given access to water during a test trial. While testing, water intake is punished by a shock through the metallic drinking spout (Millan and Brocco, 2003). Decreased drinking is an indication of higher anxiety (Millan and Brocco, 2003; Basso et al., 2011). As testing co-occurs with conditioning, it is not possible to perform within-subject control conditions and another group of animals must serve as a control group (Millan and Brocco, 2003). Furthermore, if shock intensity is too low or too high, treatment-induced anxiolytic or anxiogenic effects may be inadvertently obscured (Millan and Brocco, 2003). Motivation, nociception, thirst, and learning can all affect results, thus it is particularly useful to interpret results in conjunction with other anxiety assays.

In addition to assay-specific behavior, rodents in all paradigms demonstrating an increase in stretch-attend postures (elongating body close to the ground to survey the surroundings; Holly et al., 2016), defecation, and urination indicate greater anxiety (Seibenhener and Wooten, 2015).

## Validity of Anxiety Assays

To pharmacologically validate a rodent anxiety assay as a preclinical animal model, researchers must show that drugs that increase or decrease anxiety in humans have a similar effect in the rodent assay (Table 1).

(1) *Drugs that modulate serotonin*. Serotonin regulates mood and serotonergic drugs are commonly used to treat depression. There are serotonin agonists (like the serotonin 1A receptor agonist Buspirone), and selective serotonin reuptake inhibitors (SSRIs; like Fluoxetine) that inhibit the removal of serotonin from synapses. These drugs tend to be anxiogenic upon acute administration and anxiolytic with chronic administration in rodent tests (Stefański et al., 1992; Cole and Rodgers, 1994; Griebel et al., 1998; Silva and Brandão, 2000; Dulawa et al., 2004; Farhan and Haleem, 2016). This time course mirrors their clinical effect as acute treatments can increase anxiety in humans and typically need to be used for many weeks before they take an anxiolytic effect (Harmer et al., 2017). Side effects of SSRIs can include loss of appetite, lowered sex drive, and insomnia (Ferguson, 2001).
(2) *Benzodiazepines*. These drugs interact with GABA as a positive allosteric GABA_A_ receptor modulator (Campo-Soria et al., 2006). Benzodiazepines are commonly prescribed for acute anxiety and panic disorders, but are addictive and are not meant to be taken for more than a few weeks at a time (Lader, 2011). Benzodiazepines tend to be anxiolytic in rodent models, in agreement with their clinical use (Bruhwyler et al., 1991; Choleris et al., 2001; Sink et al., 2010; Birkett et al., 2011; Garcia et al., 2011).
(3) *Other drugs that modulate GABA*. Barbiturates are positive allosteric modulators of GABA_A_ receptors and also block excitatory ionotropic glutamate currents (Löscher and Rogawski, 2012). Barbiturates produce a sedative effect, which creates an anxiolytic phenotype in behavioral assays. They are less common than benzodiazepines in clinical settings because of their low therapeutic margin (López-Muñoz et al., 2005). Ethanol also interacts with GABA by enhancing the activity of GABA_A_ receptors (Davies, 2003). Ethanol is anxiolytic in mice, similar to alcohol consumption in humans (Gulick and Gould, 2009). In general, GABA_A_ receptor agonists tend to be anxiolytic and GABA_A_ receptor antagonists tend to be anxiogenic (Bertoglio and Carobrez, 2002; Birkett et al., 2011; Garcia et al., 2011; Miller et al., 2011; Acevedo et al., 2014; Kilic et al., 2014; Zhang et al., 2014).
(4) *Other agents*. Yohimbine is an α2 adrenergic antagonist and increases anxiety in mice (Bhattacharya et al., 1997; Braun et al., 2011; Arrant et al., 2013). It is also known to increase heart rate and anxiety in humans (Charney et al., 1983). Caffeine is another commonly used stimulant known to increase anxiety in mice (El Yacoubi et al., 2000a,b; Braun et al., 2011). It can produce anxiety in humans at high doses, and people with panic and anxiety disorders are especially sensitive to this agent (Totten and France, 1995).

**Table 1:** Effects of commonly used drugs in anxiety assays.

Drug	Class/Action	OFT center time	EPM open arm time	LDB light component time	References
Chlordiazepoxide (Diazepam)	Benzodiazepine	↑	↑	↑	Choleris et al. (2001); Birkett et al. (2011); Garcia et al. (2011)
Gabapentin	Increases GABA concentrations		↑	↑	Kilic et al. (2014); Zhang et al. (2014)
Phenobarbital	Barbiturate		↑		Bertoglio and Carobrez (2002)
Ethanol	Reduces GABA-A receptor transmission	↑	↑	↓ (High doses)—(Low doses)	Bertoglio and Carobrez (2002); Acevedo et al. (2014)
Gepirone (Chronic)	5-HT receptor agonist		↑		Silva and Brandão (2000)
Fluoxetine (Chronic)	SSRI	↑	—	↑	Silva and Brandão (2000); Dulawa et al. (2004); Farhan and Haleem (2016)
Buspirone (Chronic)	SSRI		↑*		Cole and Rodgers (1994)
Gepirone (Acute)	5-HT receptor agonist	↑	↓		Stefański et al. (1992); Silva and Brandão (2000)
Fluoxetine (Acute)	SSRI	—	↓	↓	Silva and Brandão (2000); Dulawa et al. (2004); Birkett et al. (2011)
Buspirone (Acute)	SSRI	↓*	—	—	Stefański et al. (1992); Cole and Rodgers (1994); Griebel et al. (1998)
FG 7142	Benzodiazepine inverse agonist	↓	↓	↓	Bruhwyler et al. (1991); Sink et al. (2010); Arrant et al. (2013)
Picrotoxin	GABA-A receptor antagonist		↓		Birkett et al. (2011)
Pentylenetetrazol	Reduces GABA-A receptor transmission		↓	—	Garcia et al. (2011); Miller et al. (2011)
Yohimbine	α2 adrenergic antagonist	↓	↓	↓	Bhattacharya et al. (1997); Braun et al. (2011); Arrant et al. (2013)
Caffeine	Adenosine A1/A2A receptor antagonist	↑ Locomotion	↓	↓	El Yacoubi et al. (2000a,b); Braun et al. (2011)

## Neural Circuits That Affect Anxiety

The validation of rodent anxiety assays has catalyzed the identification of neurobiological mechanisms that generate anxiety. A great deal of research implicates the amygdala, part of the phylogenetically ancient limbic system known to integrate information about external stimuli, internal body state, and pain, as a critical node in anxiety. Early studies using immediate early gene analysis and pharmacological whole-amygdala inactivation suggested increased activity in the amygdala increases anxiety (Silveira et al., 1993; Moreira et al., 2007). This was supported by evidence in humans that amygdala volume is correlated with anxiety (Machado-de-Sousa et al., 2014; Qin et al., 2014).

Furthermore, the amygdala contains distinct, non-overlapping neuronal populations that, based on their projection targets, differentially modulate behavior in anxiety assays. For example, activating basolateral amygdala neurons that project to the central lateral amygdala increases OFT center time, EPM open arm time, and EPM open arm entry (Tye et al., 2011). Importantly, this anxiolytic effect was undetectable when broadly activating basolateral amygdala neurons (Tye et al., 2011). In contrast, activating the projection from basolateral amygdala to the ventral hippocampus decreases OFT center time and EPM open arm time, indicating increased activity in this projection is anxiogenic (Felix-Ortiz et al., 2013). Overall, there is strong evidence that specific amygdalar subunits play critical and often opposing roles in modulating anxiety in rodent models and, with further delineation of their respective roles, may help establish the amygdala as an effective target for clinical intervention.

Circuit-level dissections have also helped characterize the role of the bed nuclei of the stria terminalis (BNST) in anxiety. The BNST is a sexually dimorphic limbic structure important in integrating information and is composed of many distinct nuclei. Functional magnetic resonance imaging studies indicate BNST activity in humans is correlated with anxiety (Yassa et al., 2012). In mice, researchers showed some BNST subpopulations may modulate anxiety in a functionally-opposing manner. Specifically, activation of glutamatergic BNST neurons projecting to the ventral tegmental area decreased OFT center time, indicating an enhancement in anxiety, whereas activation of the projection of GABAergic BNST neurons to the same region increased EPM open arm time, indicating a reduction in anxiety (Jennings et al., 2013).

A recent study provides strong evidence that the hippocampus, known to be critical in episodic memory and spatial information processing, also plays a key role in representing anxiogenic environments (Jimenez et al., 2018). Neurons in the ventral CA1 of the hippocampus robustly increase firing in the OFT center and EPM open arms. Further probing indicates these neurons are glutamatergic and project to the lateral hypothalamus. Strikingly, lateral hypothalamus-projecting ventral CA1 neurons appear to play a critical role in anxiety-related avoidance, as inhibition of these neurons reduces avoidance of the EPM open arms (Jimenez et al., 2018). The hippocampus has also been shown to modulate anxiety through projections to the medial prefrontal cortex (mPFC), a region that is independently known to promote avoidance in the EPM (Shah et al., 2004). Optogenetic inhibition of ventral hippocampal projections to the mPFC increases EPM open arm time, suggesting that this circuit is also critical for anxiety-related behaviors (Padilla-Coreano et al., 2016). This concurs with a study that correlates abnormal human hippocampal microstructure with a generalized anxiety disorder (Cha et al., 2016).

Other regions known to be involved in anxiety include the medial septum, the insular cortex, and the prelimbic cortex. When inactivated, the medial septum increases the time spent in the EPM open arms (Degroot and Treit, 2004). Also, insular cortex neurons have been shown to modulate anxiety differentially based on their projection targets. Specifically, inhibition of insular cortex neurons projecting to the central amygdala increases EPM open arm time, while inhibition of insular cortex neurons projecting to the nucleus accumbens decreases EPM open arm time depending on the intensity of threat (Gehrlach et al., 2019). Finally, when the approach-avoidance conflict is presented as a cost-benefit paradigm within a modified T-Maze, inhibition of striatum-projecting prelimbic cortical neurons biases rats to opt for a high-risk, high-reward option over a low-risk, low-reward option, implicating the circuit in decision making during approach-avoidance conflicts (Friedman et al., 2015). Many brain regions are involved in the formation and expression of anxiety, and researchers are only just beginning to uncover their respective functions.

## Other Perspectives

Researchers are considering other factors that may contribute to anxiety. Page et al. found a chronic increase in the activity of parvalbumin-expressing prefrontal cortex cells decreases OFT center time, but only in female mice (Page et al., 2019). Human females are more likely to develop anxiety disorders, so probing anxiety with sex specificity could have important clinical implications (Ruscio et al., 2017). Moreover, researchers are exploring how the gastrointestinal system can affect anxiety. Recent studies reveal administering certain probiotics can decrease EPM open arm time in rats, while probiotic treatment in humans can decrease neurophysiological and panic anxiety (Tillmann and Wegener, 2019; Tran et al., 2019). Patients suffering from anxiety commonly report uncomfortable interoceptive sensations in their stomach, like tightening or “dropping,” and the gut-brain emotional connection is still not fully understood (Shapiro and Nguyen, 2010). The assays described in this review may allow researchers to hone in on how brain-body interactions influence anxiety.

## Conclusion

Future studies will expand what is known about the neural underpinnings of anxiety in humans. The aim is to extend our understanding beyond the broad effects of the systemic administration of anxiety-altering agents. Rather, we must further discern which specific brain regions these drugs act on to affect anxiety, the neurochemical profiles of neuronal populations involved, how such populations modulate neural activity in downstream regions, and how they are regulated or interact with one another. Furthermore, by coupling anxiety assays with paradigms that assess other internal states, researchers may probe the basis of commonly reported symptoms that co-present with anxiety such as changes in appetite, locomotion, fatigue, and attention.

## Author Contributions

ML-V and AA determined the manuscript sections and outline. ML-V and BT wrote the manuscript. PS, BT and ML-V made the figures. AA and PS edited the manuscript. All authors contributed to the article and approved the submitted version.

## Conflict of Interest

The authors declare that the research was conducted in the absence of any commercial or financial relationships that could be construed as a potential conflict of interest.
